# 1 : 1 Ca^2+^:Cu^2+^ A‐site Order in a Ferrimagnetic Double Double Perovskite

**DOI:** 10.1002/anie.202209497

**Published:** 2022-08-26

**Authors:** Elena Solana‐Madruga, Padraig S. Kearins, Clemens Ritter, Ángel M. Arévalo‐López, J. Paul Attfield

**Affiliations:** ^1^ Centre for Science at Extreme Conditions (CSEC) and School of Chemistry University of Edinburgh Mayfield Road Edinburgh EH9 3JZ UK; ^2^ Departamento de Química Inorgánica, Facultad CC. Químicas Universidad Complutense de Madrid Spain; ^3^ Institut Laue-Langevin 38042 Grenoble Cedex France; ^4^ Univ. Lille CNRS Centrale Lille Univ. Artois UMR 8181, UCCS Unitéde Catalyse et Chimie du Solide 59000 Lille France

**Keywords:** Double Perovskites, High-Pressure Chemistry, Magnetic Properties, Solid-State Structures

## Abstract

Cation ordering in ABX_3_ perovskites is important to structural, physical and chemical properties. Here we report discovery of CaCuFeReO_6_ with the tetragonal AA′BB′O_6_ double double perovskite structure that was previously only reported for A′=Mn compositions. CaCuFeReO_6_ occurs in the same phase field as CaCu_3_Fe_2_Re_2_O_12_ demonstrating that different A‐cation ordered peroskites may be obtained in the same chemical system. CaCuFeReO_6_ has ferrimagnetic order of Fe, Re and Cu spins below *T*_C_=567 K, in contrast to Mn analogues where the Mn spins order separately at much lower temperatures. The magnetoresistance of CaCuFeReO_6_ displays low‐field “butterfly” hysteresis with an unusual change from negative to positive values as field increases. Many more AA′BB′O_6_ double double perovskites may be accessible for A′=Cu and other divalent transition metals at high pressure, so the presently known phases likely represent only the “tip of the iceberg” for this family.

## Graphical abstract

CaCuFeReO_6_ adopts the tetragonal AA′BB′O_6_ double double perovskite structure that was previously only reported for A′=Mn compositions. Ferrimagnetic order of Fe, Re and Cu spins is observed at *T*_C_=567 K and the magnetoresistance displays low‐field “butterfly” hysteresis with an unusual change from negative to positive values as field increases.

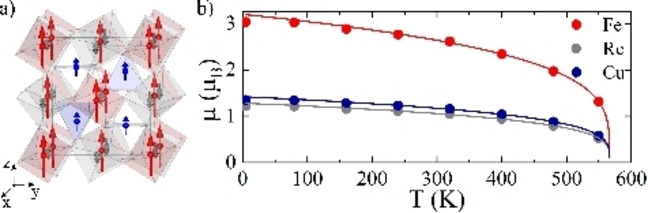

ABO_3_ perovskite oxides are important for a wide range of chemical and physical properties.[^1^, ^2^] Cation ordering within the perovskite lattice can generate emergent physical properties,^[3]^ notably in A_2_BB′O_6_ double perovskites (DPv) where rock‐salt ordering of B/B′ transition metal cations[^4^, ^5^, ^6^] leads to ferrimagnetism and large magnetoresistance in Sr_2_FeMoO_6_ and related materials.[^7^, ^8^, ^9^] More complex perovskites featuring order of alkali metal, alkaline earth or rare earth A cations with A′ transition metal ions in addition to the latter B/B′ order enable effects of the magnetic A′ transition metal ions on the B/B′ ferrimagnetism to be explored. Two such classes of compounds have been reported, both requiring high pressure synthesis to stabilise cation order based on size difference between the large A and small transition metal A′ cations as substantial tilts of the B/B′O_6_ octahedra reduce the A′ coordination number from 12 in the ideal perovskite structure to 4 in the ordered derivatives. AA′_3_B_2_B′_2_O_12_ (referred to as 1322‐type) materials have a cubic (space group *Pn*‐3) structure with 1 : 3 order of 12‐coordinate A and 4‐coordinate square‐planar A′ cations, and are formed predominantly for A′=Cu. Ferrimagnets with Curie temperatures (*T*_C_) >300 K are found for CaCu_3_B_2_B′_2_O_12_ (B/B′=Fe/Re, Fe/Os, Mn/Os)[^9^, ^10^, ^11^] and ACu_3_Fe_2_Os_2_O_12_ (A=Na, La)[^12^, ^13^] materials, with lower temperature ferri‐ or antiferro‐ magnetic orders in other analogues; CaCu_3_B_2_B′_2_O_12_ (B/B′=Ga/Sb, Cr/Sb, Fe/Sb, Fe/Nb),[^14^, ^15^, ^16^, ^17^] LaCu_3_Co_2_Re_2_O_12_ ^[18]^ and LaMn_3_Ni_2_Mn_2_O_12_.^[19]^ The latter is the only A′=Mn (and non‐Cu) 1322 phase reported to date.

The second group of A/A′ and B/B′ ordered perovskites have AA′BB′O_6_ stoichiometry and are labelled double double (or doubly ordered) perovskites (DDPv's) following the “double perovskite” description for AA′B_2_O_6_ or A_2_BB′O_6_ materials with cation order at only one type of site.^[3]^ They have tetragonal *P*4_2_/*n* symmetry with columnar ordering of 10‐coordinate A and 4‐coordinate A′ cations, and all previously reported materials are based on A′=Mn. RMnMnB′O_6_ (R=rare earth) series are reported for B′=Sb[^20^, ^21^] and Ta,^[22]^ and many CaMnBB′O_6_ materials (B=Mn, Fe, Co, Ni for B′=Re,[^23^, ^24^, ^25^] and B/B′=Fe/Ta,^[26]^ Cr/Sb and Fe/Sb^[27]^) were also synthesised at high temperatures and pressures. High temperature ferrimagnetism was reported in CaMnFeReO_6_ (*T*_C_=500 K) and also a Cu‐doped derivative of nominal composition Ca(Mn_0.5_Cu_0.5_)FeReO_6_ (*T*_C_=560 K), with an unusual switch between negative and positive magnetoresistances in the latter at low temperatures.^[23]^ The latter material suggests that DDPv's stabilised by A/Cu ordering, without any Mn content, may be accessible. This offers the exciting possibility of being able to stabilise AA′_3_B_2_B′_2_O_12_ (1322) and AA′BB′O_6_ (DDPv) cation ordered perovskites in the same A−A′(=Cu)‐B−B′ chemical system for the first time, and hence compare effects of the 1 : 3 and 1 : 1 A : Cu orders on the magnetic and electronic properties of the B/B′ sublattice. Here we report synthesis of a Mn‐free, high‐*T*_C_ DDPv material CaCuFeReO_6_ and comparison of properties to the previously reported CaCu_3_Fe_2_Re_2_O_12_.^[9]^

Samples of nominal composition CaCuFeReO_6_ were heated under pressures of 10–15.5 GPa using stoichiometric mixtures of Ca_2_Fe_2_O_5_, ReO_2_ and CuO in a Walker‐type module as described in Supporting Information. A component with the *P*4_2_/*n* DDPv type structure was identified by powder X‐ray diffraction within multiphase products. Optimum samples were synthesised at 15.5 GPa and 1400 °C and were characterised by powder synchrotron X‐ray diffraction at 300 K, neutron diffraction patterns from 5 to 550 K, and magnetic and electronic transport measurements (details in Supporting Information).

The crystal structure of CaCuFeReO_6_ was refined using synchrotron X‐ray^[28]^ and neutron powder diffraction profiles. 6.8(2)% Fe/Re antisite disorder was found from the X‐ray fit (full results are in Supporting Information) and this value was fixed in all neutron refinements. The A′=Cu cation columns have two inequivalent sites with tetrahedral (Cu1) and square planar (Cu2) coordinations. Refinement against powder neutron data (Figure 1) showed that 29(1) and 0(1)% of Fe was substituted at the Cu1 and Cu2 sites, respectively, so their occupancies were fixed as 70 %Cu/30 %Fe and 100 %Cu in subsequent variable temperature neutron fits.

**Figure 1:**
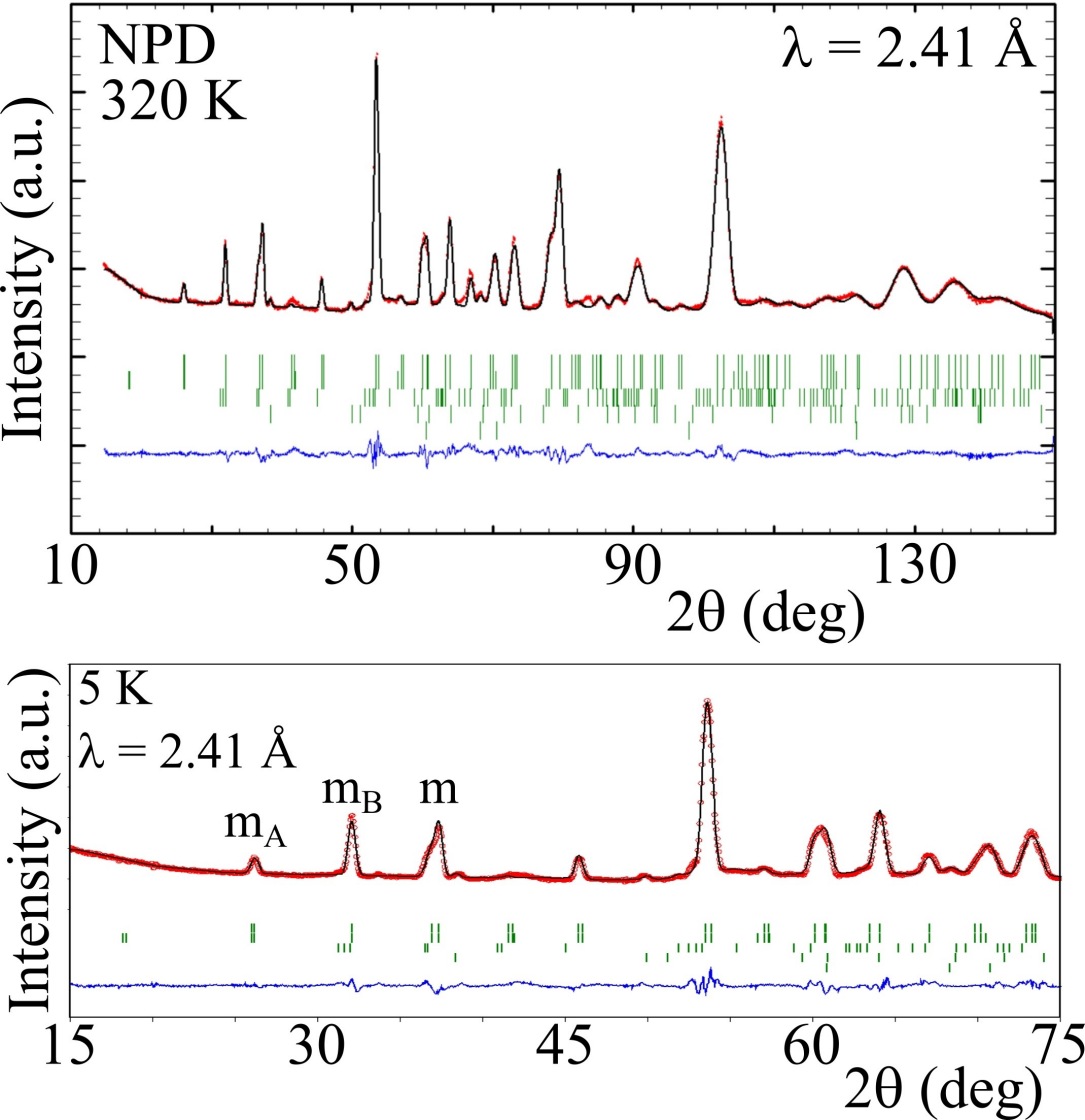
Rietveld fits of the DDPv structure of CaCuFeReO_6_ against NPD data at 320 K showing the full range of data (top) and at 5 K showing the low‐angle magnetic peaks (bottom), labelled m_A_/m_B_/m to show peaks from spins at A/B/(A and B) type cation sites. Markers from top to bottom show CaCuFeReO_6_ DDPv (82(1) w.t. %), CaCuFeReO_6_ DPv (5.3(3) %), ReO_2_ (7.4(4) %) and Re (4.8(3) %).

Magnetic diffraction peaks from CaCuFeReO_6_ (Figure 1) were observed in all neutron diffraction patterns from 5 to 550 K. Peaks from ordered moments at both A‐site Cu and B/B′ Fe/Re are observed. The magnetic peaks are indexed by propagation vector [0 0 0] and good fits were obtained from a ferrimagnetic model with spins parallel to the *c*‐axis (Figure 2). The small Re spin was found to be unstable in initial magnetic refinements and was constrained to be antiparallel to the Fe moment as done in previous refinements of Mn‐based CaA′FeReO_6_ materials.^[23]^ The two Cu site spins were both found to be parallel to the Fe spin and were constrained to be equal in the final refinements. Results of the refinement of crystal and magnetic structures against powder neutron diffraction data are shown in Figures 1 and 2 and are tabulated in Supporting Information.

**Figure 2:**
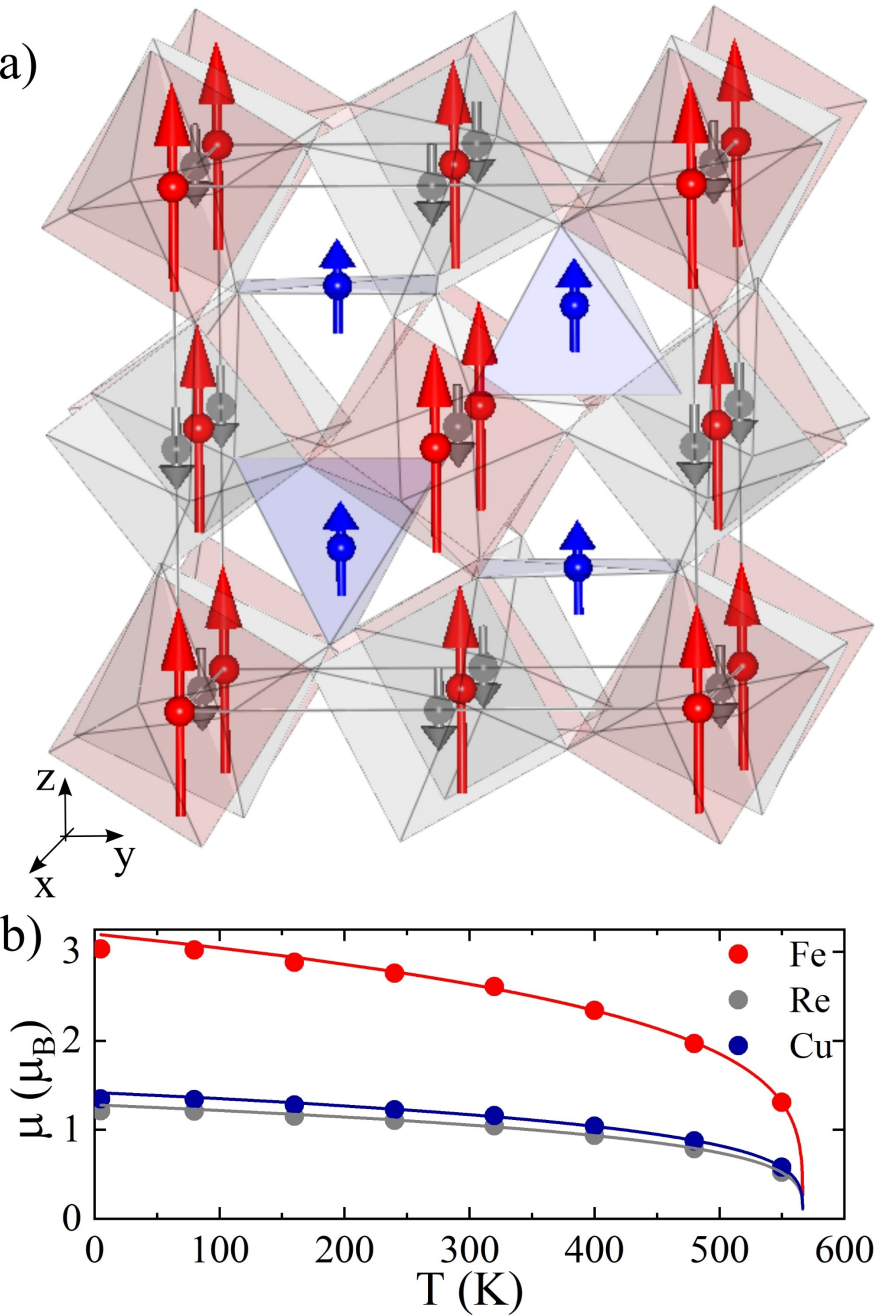
a) Refined crystal and magnetic structures for CaCuFeReO_6_ with Fe, Re and Cu magnetic moments as red, grey and blue arrows, respectively. b) Refined magnetic moments vs. temperature with critical functions fitted in the *T*_C_/2<*T*<*T*_C_ temperature range.

The crystal structure refinements demonstrate that the present CaCuFeReO_6_ sample is slightly off‐stoichiometric with refined composition CaCu_0.85_Fe_1.15_ReO_6_ and also with 7 % Fe/Re antisite disorder. Fe shows a clear preference for substituting at the tetrahedral (Cu1) rather that the square planar (Cu2) site. Nevertheless the material shows a very high degree of order of four different metals over five cation sites, and demonstrates that the double double perovskite arrangement, previously reported only for Mn‐based materials, can be stabilised at pressure by Ca/Cu ordering over A/A′ columns.

Bond valence sum calculations using the 320 K neutron bond lengths give BVS values Ca=2.3, Cu1=1.6, Cu 2=1.5, Fe=2.6 and Re=5.1. These are consistent with the ideal valence distribution Ca^2+^Cu^2+^Fe^3+^Re^5+^O_6_ which is analogous to those in related materials such as Ca^2+^Mn^2+^Fe^3+^Re^5+^O_6_,^[23]^ allowing for disorder at some sites and structural strain induced by high pressure synthesis. Octahedral tilt angles calculated from Fe−O−Re angles along the *c* axis (ϕ=19.3(4)°) and within the *ab* plane (*θ*=17.4(2)°) are within the range of 15 to 19.5° for Mn‐based analogues^[20]^ and thus demonstrate that these substantial tilts are essential to stabilisation of this DDPv structure type.

The same ferrimagnetically ordered spin structure (Figure 2a) was observed in CaCuFeReO_6_ from 5 to 550 K, and thermal variations of the refined magnetic moments are shown in Figure 2b. The two independently refined magnetic moments (Fe/Re and Cu1/Cu2) both follow the same critical dependence ≈[1−(*T*/*T*_C_)]^β^ with fitted *T*_C_=566.70(3) K and *β*=0.254(2). The latter is close to the ideal mean field value of *β*=0.25 for a tricritical transition, on the cusp between first and second order behaviour. This may reflect the complex network of interactions between four distinct spin sublattices. Ordered moments at 5 K of 3.0 and 1.2 μ_B_ for Fe^3+^ and Re^5+^ (refined in a 5 : 2 ratio) are reduced from ideal values of 5 and 2 μ_B_ reflecting B/B′ antisite disorder and spin‐orbit coupling for Re^5+^. However the average moment at 5 K for the Cu sites of 1.4 μ_B_ exceeds the ideal 1 μ_B_ value for Cu^2+^ showing that the Fe spins substituted at the Cu sites are also aligned. Mössbauer spectroscopy would be useful for further characterisation of the Fe states at the Cu sites.

Physical properties of CaCuFeReO_6_ were explored through magnetisation and resistivity measurements in zero and applied fields. Magnetic susceptibility, shown in Figure 3a, reveals a single Curie transition. The estimated *T*_C_=551 K, from extrapolation of the steepest negative χ–*T* slope to χ=0, is in agreement with the value of *T*_C_=567 K from fitting the zero field neutron moments above. A Curie–Weiss fit to data at 600–625 K gives realistic results despite the narrow fitting range; an effective paramagnetic moment of μ_eff_=6.52 μ_B_/f.u., close to the spin‐only estimate of 6.78 μ_B_/f.u., and a Weiss temperature of *θ*=522 K, comparable to *T*_C_ and showing that 3D ferrimagnetic order occurs with little frustration. Magnetisation‐field hysteresis loops confirm the ferrimagnetic order, with a saturated magnetic moment of 4 μ_B_/f.u. (Figure 3b) at 2 and 100 K identical to the value expected for order of *S*=1/2 ↑ Cu^2+^, *S*=5/2 ↑ Fe^3+^, and *S*=1 ↓ Re^5+^ spins, confirming the arrangement derived from neutron diffraction. The saturated moment decreases to 2.7 μ_B_ at 400 K (see Supporting Information). Small coercive fields of 120 and 97 mT are observed at 2 and 100 K respectively, reflecting the weak anisotropy of the tetragonal DDPv structure.

**Figure 3:**
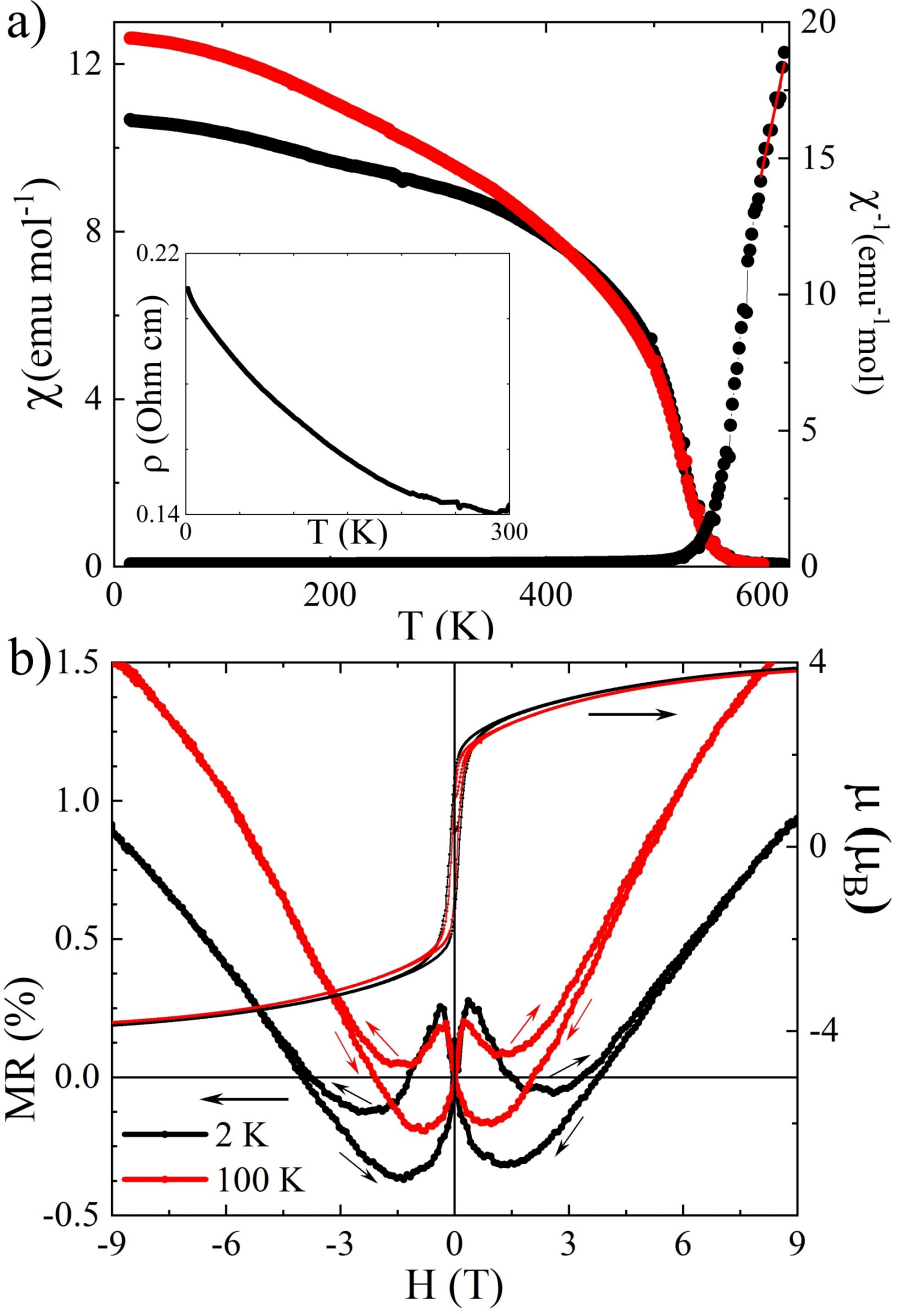
a) FC‐ZFC (red‐black) magnetic susceptibility (left) and inverse (right) of CaCuFeReO_6_. Inset shows T‐dependence of resistivity. b) Hysteresis loops and butterfly‐shaped magnetoresistance at 2 and 100 K.

The resistivity of a ceramic pellet of CaCuFeReO_6_ is 140 mΩ cm at room temperature increasing to 210 mΩ cm to 4 K (Figure 2a). This change corresponds to an energy gap of ≈1 meV which is unrealistically small for intrinsic semiconducting behaviour suggesting that grain boundary resistances mask an underlying metallic conductivity. Magnetoresistance (MR) values in Figure 3b are small, most likely due to the Fe/Re antisite disorder, but show interesting variations with field. The negative low field effect is a typical spin‐valve‐type magnetoresistance due to the intergrain tunneling of spin‐polarized conduction carriers, as observed in Sr_2_FeMoO_6_ and other ferromagnetic double perovskites. However a positive MR effect dominates at high fields. A similar switch from negative to positive MR was reported in the related Ca(Mn_0.5_Cu_0.5_)FeReO_6_ DDPv^[23]^ and in the DPv Mn_2_FeReO_6_[^29^, ^30^] due to canting of Fe and Re spins arising from frustration of their order with respect to the Mn spins as field increases, and a similar mechanism may operate here due to their interactions with the Cu site spins. Low field “butterfly” MR hysteresis curves are observed but the hysteresis in MR differs slightly from that in the magnetization as the peak‐to‐peak MR separations do not coincide with the coercive fields. The behavior is also observed in Sr_2_FeMoO_6_ and the 1322‐type analogue to CaCuFeReO_6,_ CaCu_3_Fe_2_Re_2_O_12_,^[9]^ and for epitaxial Fe_3_O_4_ films and NiCo_2_O_4_[^31^, ^32^]

CaCuFeReO_6_ is an important discovery in the field of cation‐ordered perovskites as it demonstrates that the *P*4_2_/*n* AA′BB′O_6_ double double perovskite (DDPv) structure already reported for many RMnBB′O_6_ and CaMnBB′O_6_ materials can also be stabilised for A′=Cu. CaCuFeReO_6_ is the first reported Mn‐free DDPv. Synthesis of CaCuFeReO_6_ has proved more challenging than for the CaMnFeReO_6_ analogue.^[23]^ The present neutron sample of CaCuFeReO_6_ was recovered from 15.5 GPa and 1400 °C, has 82 % phase purity, with 7 % Fe/Re antisite disorder and 30 % Fe substituted at one of the Cu sites, whereas CaMnFeReO_6_, prepared at 10 GPa and 1400 °C was >99 % phase pure with 3 % Fe/Re antisite disorder and 8 and 16 % Fe at the two Cu sites. Nevertheless this study indicates that increasing pressure significantly above 10 GPa may enable many more AA′BB′O_6_ DDPvs to be synthesised for A′=Cu and perhaps other divalent A′=Fe, Co, Ni… cations, so the presently known phases likely represent only the “tip of the iceberg” of DDPv materials.

Interesting differences between the physical properties of CaCuFeReO_6_ and CaMnFeReO_6_ are apparent. Both have high *T*_C_’s for ferrimagnetic Fe/Re spin order, of 567 and 500 K respectively, typical for A_2_FeReO_6_ double perovskites, but the Cu spins also order at *T*_C_ in CaCuFeReO_6_ whereas the A‐site Mn spins in CaMnFeReO_6_ order at a separate transition at *T*_A_=70 K.^[9]^ Order of Mn^2+^ spins at *T*_A_=75 K, well below *T*_C_=520 K, is also seen in the double perovskite Mn_2_FeReO_6_.[^28^, ^29^] These differences evidence stronger Fe/Re−O−A′ superexchange for A′=Cu compared to A′=Mn, and perhaps also a shift in relative values of the various exchange interactions so that A′ spins in CaCuFeReO_6_ are less magnetically frustrated. CaMnFeReO_6_ has negative MR diverging to large values at low temperature and high field (−32 % at 20 K and 7 T), typical for ferrimagnetic double perovskites like Sr_2_FeMoO_6_,^[7]^ whereas CaCuFeReO_6_ shows a switch from negative to positive MR with increasing field. A similar switch in Mn_2_FeReO_6_ results from increased canting of Fe and Re spins due to frustration of their order with respect to the Mn spins as field increases. High field neutron diffraction experiments would be needed to verify the same effect in CaCuFeReO_6_. It is also interesting to compare the mixed DDPv Ca(Mn_0.5_Cu_0.5_)FeReO_6_ ^[23]^ to CaA′FeReO_6_ (A′=Cu and Mn). Ca(Mn_0.5_Cu_0.5_)FeReO_6_ has *T*_C_=560 K and a separate ordering transition at *T*_A_=150 K unlike in CaCuFeReO_6_, but the increase relative to *T*_A_=70 K in CaMnFeReO_6_ demonstrates the effect of Cu in strengthening Fe/Re‐O−A′ superexchange. However Ca(Mn_0.5_Cu_0.5_)FeReO_6_ displays a switch from negative to positive MR on warming, as seen in CaCuFeReO_6_ (e.g. by comparing 2 and 100 K MR values in a 3 T field in Figure 3b) but not in CaMnFeReO_6_. Such complex MR behaviour is therefore not directly linked to the presence (or absence) of a distinct ordering transition of A‐site spins, but rather to field effects on competing A′−O−B, A′−O−B′ and B−O−B′ interactions within AA′BB′O_6_ DDPvs with ordered A′, B and B′ spin sublattices.

Formation of CaCuFeReO_6_ is also important because a 1322 type phase CaCu_3_Fe_2_Re_2_O_12_ (written here as Ca_1/2_Cu_3/2_FeReO_6_ for comparison) is also known and so this is the first demonstration that two different A‐cation ordered perovskites can be prepared within the same chemical system. Comparison of their properties is again instructive. CaCuFeReO_6_ and Ca_1/2_Cu_3/2_FeReO_6_ have similar *T*_C_’s of 567 and 560 K with Cu spins ordering parallel to Fe spins at *T*_C_ leading to large saturated magnetisations of 4.0 and 4.35 μ_B_ respectively, the latter value reflecting the extra 0.5 Cu^2+^ spin per double perovskite unit. Both materials have apparent metallic conductivity but with resistive grain boundaries in ceramic samples. A high spin‐polarization of conduction electrons was reported for Ca_1/2_Cu_3/2_FeReO_6_ and both materials display low‐field “butterfly” spin‐valve MR effects although the magnitude of MR is small. However, Ca_1/2_Cu_3/2_FeReO_6_ has negative MR while CaCuFeReO_6_ shows an unusual switch to positive values at high field. Exploration and comparison of DDPv, 1322 type, and perhaps other types of A and B‐site ordered perovskites in mixed transition metal oxide systems is likely to lead to discovery of spintronic and other types of interesting property.

In conclusion, discovery of CaCuFeReO_6_ marks an important milestone in development of A‐ and B‐ cation ordered perovskites, as it demonstrates that the tetragonal AA′BB′O_6_ double double perovskite structure, previously only reported for A′=Mn, can be stabilised by Cu and perhaps other metals at pressures above 10 GPa. CaCuFeReO_6_ occurs in the same phase field as 1322‐type CaCu_3_Fe_2_Re_2_O_12_ demonstrating that different A‐cation ordering types may be obtained in the same chemical system. Ferrimagnetic Fe (up), Re (down) and Cu (up) spin order occurs in CaCuFeReO_6_ below *T*_C_=567 K and magnetoresistance shows low‐field spin‐valve‐type butterfly hysteresis with an unusual change from negative to positive values as field increases.

## Conflict of interest

The authors declare no conflict of interest.

## Supporting information

As a service to our authors and readers, this journal provides supporting information supplied by the authors. Such materials are peer reviewed and may be re‐organized for online delivery, but are not copy‐edited or typeset. Technical support issues arising from supporting information (other than missing files) should be addressed to the authors.

Supporting Information

Supporting Information

Supporting Information

Supporting Information

Supporting Information

Supporting Information

Supporting Information

Supporting Information

Supporting Information

Supporting Information

Supporting Information

## Data Availability

The data that support the findings of this study are available in the Supporting Information of this article.
