# Supplementary material for: 1 : 1 Ca2+:Cu2+ A‐site Order in a Ferrimagnetic Double Double Perovskite
Source: Angew Chem Int Ed Engl. 2022 Aug 26;61(40):e202209497. doi: 10.1002/anie.202209497 (PMC9805228; doi:10.1002/anie.202209497)
Supplement: Supplementary file 1 — Supporting Information [file ANIE-61-0-s007.pdf]

Angewandte Chemie
Eine Zeitschrift der Gesellschaft Deutscher Chemiker

Supporting Information

1:1 Ca^{2+}:Cu^{2+} A-site Order in a Ferrimagnetic Double Double Perovskite

E. Solana-Madruga, P. S. Kearins, C. Ritter, Á. M. Arévalo-López, J. P. Attfield**

WILEY-VCH

Supporting Information

Experimental Methods

HPHT Synthesis

Synthesis of DDPv $CaCuFeReO_6$ was performed under HPHT (high pressure high temperature) conditions using a Walker type module. The precursor mixture containing stoichiometric proportions of $Ca_2Fe_2O_5$, ReO_2 and CuO was ground in acetone using an agate mortar and pestle and packed into a Pt capsule, pressed under pressures of 10 - 15.5 GPa and heated. After 20 minutes under target temperature, the sample was quenched and pressure was slowly released. A single HPHT reaction was carried out for each 8 mm^3 sample. A DDPv phase was observed in products synthesised at pressures of 10 - 15.5 GPa and temperatures 1000 – 1400 ºC. Phase purity was found to improve for higher pressure and synthesis conditions were optimised at 15.5 GPa and 1400 ºC based on laboratory powder XRD (X-ray diffraction). The main secondary phase found from XRD was ReO_2 and high temperature conditions were needed to minimise this. Minor amounts of a double perovskite (DPv) phase (nominally $(Ca_{0.5}Cu_{0.5})_2FeReO_6$) were not observed in lab XRD but are evident in the synchrotron XRD profiles. The presence of this phase suggests that both DPv and DDPv polymorphs of $CaCuFeReO_6$ may be accessible, as recently observed for some Mn-based analogues. [1] The highest DDPv phase purity was 90% for the sample studied by synchrotron XRD (Fig. S1) but the neutron sample made by combining several synthesis products had a lower purity of 82% (Fig. 1).

Crystallographic characterisation

Room temperature synchrotron X-ray diffraction (SXRD) data were collected at I11 beamline at the Diamond facility using wavelength $\lambda = 0.82651$ Å. Neutron powder diffraction (NPD) data were collected at the ILL D20 beamline on a 50 mg sample combining several synthesis products. Profiles with $\lambda = 2.41$ Å were collected at 5, 80, 160, 240, 320, 400, 480 and 550 K using a cryofurnace. An additional pattern was collected at 538 K in high resolution mode with $\lambda = 1.54$ Å. Magnetic symmetry analysis was carried out using BASIREPS [2] and X-ray and neutron diffraction patterns were refined through the Rietveld method using the Fullprof Suite.[3]

Structure refinements included investigation of possible transition metal cation disorder in the $P4_2/n$ DDPv type $AA'BB'O_6$ structure. Ca and O sites were found to be fully occupied. Antisite exchange between Fe and Re octahedral sites could be refined as 6.8(1)% from the SXRD refinement (Table S1 and Fig. S1), given their different atomic numbers $Z_{Fe} = 26$ and $Z_{Re} = 75$. The resulting values were fixed for NPD refinements due to their poor scattering length contrast: 9.54 and 9.2 fm for Fe and Re respectively. The large contrast between neutron scattering lengths for Fe and Cu (7.718 fm) allowed accurate Fe occupancies at Cu sites to be refined from high resolution NPD data (Tables S2 and S3, and Figure S2), showing that 28(2)% of the tetrahedral Cu1 site is occupied by Fe, while the square-planar Cu2 sites contain no Fe within error. A fit to the 320 K NPD data collected with 2.41 Å is presented in Table S2 as the closest neutron refinement to room temperature. Similar site occupancies were confirmed for Cu sites, while thermal factors could be refined independently for Ca, Cu, B/B' and O sites.

All magnetic peaks can be indexed using propagation vector k = [0 0 0] and the magnetic symmetry analysis is the same as those of previously reported related DDPvs, such as $CaMnFeReO_6$ [4]. All magnetic moments follow Irep Γ_1, with collinear alignment along the *c* axis into a ferrimagnetic structure with Re spins opposed to Fe and Cu. For stability of the refinements, magnitudes of Fe and Re moments were constrained in a 5:2 ratio, based on their spins of S = 5/2 and 1 respectively, allowing independent refinement of the Cu spin (assumed to be the same at Cu1 and Cu2 sites). All cation occupancies were fixed during the final magnetic refinements, as described in the main text, enabling realistic thermal factors and magnetic moments to be refined against $\lambda = 2.41$ Å NPD profiles at all temperatures. Results are in Tables S4 and S5. The resulting moments follow a critical law as detailed in the main text.

Magnetic characterisation

Bulk magnetic properties were measured using a Quantum Design MPMS and a 9T PPMS-Dynacool magnetometers. Magnetic susceptibility was measured in ZFC-FC mode using a 1000 Oe field between 2 and 625 K. The oven option was used for MPMS high temperature data collection. Data are consistent for the common temperature range, allowing a reliable data analysis for bulk magnetic behaviour. Hysteresis loops were collected at several temperatures from 2 to 400 K for magnetic fields ranging -9 to 9 T using a PPMS (Figure S3). Magnetoresistance was measured on a HPHT sample pellet 0.56 mm thick and with a 0.755 mm radius, using two contacts. R vs H was measured at 2 and 100 K for the -9 to 9 T field range.

Supporting tables

Table S1. Main structural details as refined against 300 K SXRD data for DDPv $CaCuFeReO_6$. Space group *P4₂/n* and cell parameters a = 7.5344(2) Å and c = 7.6377(3) Å. Agreement factors R_p = 14.7%, R_{wp} = 20.6 %, R_B = 13.8%, R_f = 10.4%, χ^2 = 8.82.

Site	x	y	z	Occ	B_{iso} (Å^2)
Ca (4e)	0.25	0.75	0.770(3)	1	0.7(1)
Cu1 (2a)	0.75	0.75	0.75	1	0.9(1)
Cu2 (2b)	0.25	0.25	0.75	1	0.9(1)
Fe (4c) / Re	0.0	0.5	0.5	0.932 / 0.068(1)	0.2(1)
Re (4d) / Fe	0.0	0.0	0.5	0.932 / 0.068(1)	0.2(1)
O1 (8g)	-0.102(7)	0.542(5)	0.254(3)	1	0.9(2)
O2 (8g)	-0.255(1)	-0.025(5)	0.594(4)	1	0.9(2)
O3 (8g)	-0.238(3)	0.060(3)	-0.040(5)	1	0.9(2)

Table S2. Atomic positions and main interatomic distances and angles of $CaCuFeReO_6$ as refined against 320 K NPD data (*P4₂/n*; *a* = 7.5381(2) Å and *c* = 7.6327(4) Å; residuals R_p = 2.87%, R_{wp} = 3.97%, R_B = 4.14%, R_f = 3.32%, R_{mag} = 5.86%, χ^2 = 11.9). Refined magnetic moments; μ_{Fe} = 2.61(2) μ_B, μ_{Re} = -1.04(1) μ_B, μ_{Cu} = 1.18(1) μ_B

Site	x	y	z	Occ	B_{iso} (Å^2)
Ca (4e)	0.25	0.75	0.769(1)	1	0.2(2)
Cu1 (2a) /Fe	0.75	0.75	0.75	0.71/ 0.29(1)	1.1(1)
Cu2 (2b) /Fe	0.25	0.25	0.75	1.00/ 0.00(1)	1.1
Fe (4c) /Re	0.0	0.5	0.5	0.932/ 0.068	1.19(3)
Re (4d) /Fe	0.0	0.0	0.5	0.932/ 0.068	1.19
O1 (8g)	-0.044(1)	0.576(1)	0.247(2)	1	0.83(5)
O2 (8g)	-0.239(2)	-0.042(1)	0.580(1)	1	0.83
O3 (8g)	-0.265(1)	0.058(1)	-0.034(1)	1	0.83

Bond A-O	Length (Å)	Bond B-O	Length (Å)
(Ca-O1)x2	2.914(7)	(Fe-O1)x2	2.04(1)
(Ca-O1)x2	2.585(7)	(Fe-O2)x2	2.09(1)
(Ca-O2)x2	2.465(8)	(Fe-O3)x2	2.06(1)
(Ca-O3)x2	2.46(1)	<Fe-O>	2.06(1)
(Ca-O3)x2	2.36(1)	(Re-O1)x2	2.00(1)
<Ca-O>	2.56(1)	(Re-O2)x2	1.93(1)
(Cu1-O2)x4	2.040(6)	(Re-O3)x2	1.84(1)
(Cu2-O1)x4	2.029(7)	<Re-O>	1.92(1)
Bond angles (°)		<Fe-O2-Re>	139.8(3)
<Fe-O1-Re>	141.5(4)	<Fe-O3-Re>	150.5(4)

Table S3. Main structural details as refined against 538 K high resolution NPD data for DDPv_$CaCuFeReO_6$ using λ = 1.54 Å. Space group $P4_2/n$ and cell parameters a = 7.5386(1) Å and c = 7.6413(3) Å. Agreement factors R_p = 3.88%, R_{wp} = 5.05 %, R_B = 8.91%, R_f = 6.95%, χ^2 = 13.6%. (R_{mag} = 11.2%). μ_{Fe} = 1.2(1)μ_B, μ_{Re} = 0.48(4)μ_B, μ_{Cu} = 0.54(2)μ_B.

Site	x	y	z	Occ	B_{iso} (Å^2)
Ca (4e)	0.25	0.75	0.769(1)	1	1.5(1)
Cu1 (2a) / Fe	0.75	0.75	0.75	0.72 / 0.28 (2)	0.9(1)
Cu2 (2b) / Fe	0.25	0.25	0.75	1.00 / 0.00 (2)	0.9(1)
Fe (4c) / Re	0.0	0.5	0.5	0.932 / 0.068	0.48(3)
Re (4d) / Fe	0.0	0.0	0.5	0.932 / 0.068	0.48(3)
O1 (8g)	-0.0529(8)	0.5715(8)	0.248(2)	1	0.70(3)
O2 (8g)	-0.267(1)	-0.0435(7)	0.5783(5)	1	0.70(3)
O3 (8g)	-0.242(1)	0.0525(6)	-0.0367(5)	1	0.70(3)

Table S4. Structural model as refined against 5 K NPD data for DDPv_$CaCuFeReO_6$ using λ = 2.41 Å. Space group $P4_2/n$ and cell parameters a = 7.5295(1) Å and c = 7.6169(3) Å. Agreement factors R_p = 2.90%, R_{wp} = 3.94 %, R_B = 3.70%, R_f = 3.44%, χ^2 = 11.0, R_{mag} = 3.00%. μ_{Fe} = 3.03(2) μ_B, μ_{Re} = 1.213(9) μ_B, μ_{Cu} = 1.37(1) μ_B

Site	x	y	z	Occ	B_{iso} (Å^2)
Ca (4e)	0.25	0.75	0.777(1)	1	1.9(2)
Cu1 (2a) / Fe	0.75	0.75	0.75	0.7 / 0.3	0.6(1)
Cu2 (2b)	0.25	0.25	0.75	1	0.6(1)
Fe (4c) / Re	0.0	0.5	0.5	0.932 / 0.068	1.30(3)
Re (4d) / Fe	0.0	0.0	0.5	0.932 / 0.068	1.30(3)
O1 (8g)	-0.0456(1)	0.574(1)	0.247(3)	1	1.43(5)
O2 (8g)	-0.243(3)	-0.0457(7)	0.5804(6)	1	1.43(5)
O3 (8g)	-0.255(2)	0.0590(6)	-0.0335(7)	1	1.43(5)

Table S5. Main structural details as refined against variable temperature NPD data for DDPv_$CaCuFeReO_6$ using λ = 2.41 Å. Space group $P4_2/n$.

Parameter	80 K	160 K	240 K	400 K	480 K	550 K
Ca z	0.777(1)	0.779(1)	0.778(1)	0.776(1)	0.773(1)	0.774(2)
O1 x	-0.045(1)	-0.046(1)	-0.0444(9)	-0.0447(9)	-0.0437(9)	-0.044(1)
O1 y	0.5736(9)	0.573(1)	0.5756(9)	0.5761(9)	0.5769(9)	0.5774(9)
O1 z	0.247(3)	0.248(3)	0.247(2)	0.249(2)	0.247(2)	0.246(2)
O2 x	-0.244(3)	-0.244(3)	-0.238(2)	-0.239(2)	-0.240(2)	-0.240(2)
O2 y	-0.0456(7)	-0.0443(7)	-0.0430(7)	-0.0413(7)	-0.0393(7)	-0.0393(8)
O2 z	0.5802(5)	0.5795(5)	0.5802(5)	0.5792(6)	0.5784(6)	0.5779(7)
O3 x	-0.255(2)	-0.257(2)	-0.263(1)	-0.268(1)	-0.269(1)	-0.270(1)
O3 y	0.0590(6)	0.0589(4)	0.0571(6)	0.0555(7)	0.0559(6)	0.0552(8)
O3 z	-0.0335(7)	-0.0335(7)	-0.0332(7)	-0.0350(7)	-0.0357(7)	-0.0356(8)
a (Å)	7.5297(1)	7.5317(2)	7.5344(2)	7.5429(2)	7.5477(2)	7.5526(2)
c (Å)	7.6177(3)	7.6215(3)	7.6265(4)	7.6403(4)	7.6480(4)	7.6548(4)
μ_{Fe} (μ_B)	3.02(2)	2.88(2)	2.76(2)	2.34(3)	1.97(3)	1.31(5)
μ_{Re} (μ_B)	1.205(9)	1.150(9)	1.10(1)	0.94(1)	-0.79(1)	-0.52(2)
μ_{Cu} (μ_B)	1.36(1)	1.30(1)	1.25(1)	1.06(1)	0.89(1)	0.59(2)
R_p (%)	2.84	2.78	2.77	3.04	3.29	3.45
R_{wp} (%)	3.88	3.85	3.90	4.19	4.50	4.78
R_B (%)	3.83	3.88	4.36	4.92	5.20	5.47
R_f (%)	3.41	3.52	3.71	4.09	4.43	4.61
χ^2	10.7	10.6	11.3	13.4	15.6	17.7
R_{mag} (%)	3.96	4.04	6.20	7.29	9.29	12.1

WILEY-VCH

Supplementary figures

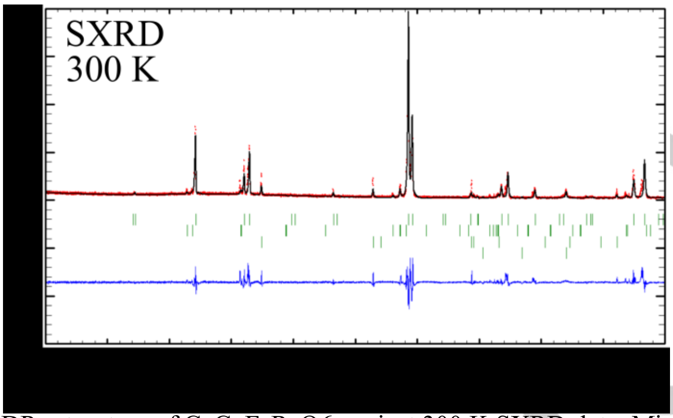

Figure S1. Rietveld fit of the DDPv structure of CaCuFeReO6 against 300 K SXRD data. Minor amounts of secondary $(CaCu)_2FeReO_6$ DPv (5.1(2)%), ReO_2 (3.7(1)%) and Re (1.29(6)%) are included.

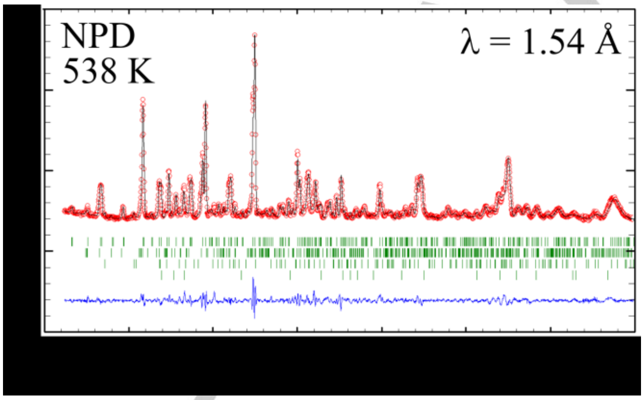

Figure S2. Rietveld fit of the DDPv structure of $CaCuFeReO_6$ against high resolution NPD data collected at 538 K using λ = 1.54 Å.

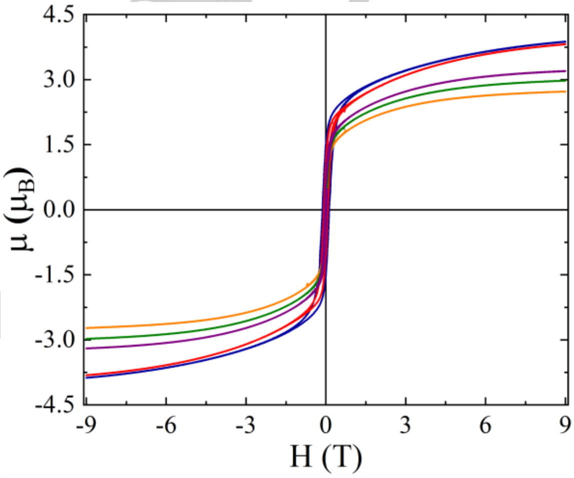

Figure S3. Magnetisation hysteresis loops at 2, 100, 300, 350 and 400 K in blue, red, purple, green and orange respectively.

References:
[1] K. Ji, K. N. Alharbi, E. Solana-Madruga, G. T. Moyo, C. Ritter, J. P. Attfield, *Angew. Chem. Int. Ed.* 2021, **60**, 41, 22248-22252.
[2] J. Rodríguez-Carvajal, *Program included FullProf Suite, version July-2010, ILL* **2010**.
[3] J. Rodríguez-Carvajal, *Physica B: Condensed Matter* **1993**, *192*, 55-69.
[4] G. M. McNally, Á. M. Arévalo-López, P. Kearins, F. Orlandi, P. Manuel, J. P. Attfield, *Chem. Mater.* 2017, **29**, 20, 8870–8874.
